# CancerClock: A DNA Methylation Age Predictor to Identify and Characterize Aging Clock in Pan-Cancer

**DOI:** 10.3389/fbioe.2019.00388

**Published:** 2019-12-04

**Authors:** Tongtong Zhu, Yue Gao, Junwei Wang, Xin Li, Shipeng Shang, Yanxia Wang, Shuang Guo, Hanxiao Zhou, Hongjia Liu, Dailin Sun, Hong Chen, Li Wang, Shangwei Ning

**Affiliations:** ^1^College of Bioinformatics Science and Technology, Harbin Medical University, Harbin, China; ^2^Department of Respiratory Medicine, The Second Affiliated Hospital of Harbin Medical University, Harbin, China

**Keywords:** chronological age, methylation age, pan-cancer, LASSO, survival

## Abstract

Many biological indicators related to chronological age have been proposed. Recent studies found that epigenetic clock or DNA methylation age is highly correlated with chronological age. In particular, a significant difference between DNA methylation age (m-age) and chronological age was observed in cancers. However, the prediction and characterization of m-age in pan-cancer remains an explored area. In this study, 1,631 age-related methylation sites in normal tissues were discovered and analyzed. A comprehensive computational model named CancerClock was constructed to predict the m-age for normal samples based on methylation levels of the extracted methylation sites. LASSO linear regression model was used to screen and train the CancerClock model in normal tissues. The accuracy of CancerClock has proved to be 81%, and the correlation value between chronological age and m-age was 0.939 (*P* < 0.01). Next, CancerClock was used to evaluate the difference between m-age and chronological age for 33 cancer types from TCGA. There were significant differences between predicted m-age and chronological age in large number of cancer samples. These cancer samples were defined as “age-related cancer samples” and they have some differential methylation sites. The differences between predicted m-age and chronological age may contribute to cancer development. Some of these differential methylation sites were associated with cancer survival. CancerClock provided assistance in estimating the m-age in normal and cancer samples. The changes between m-age and chronological age may improve the diagnosis and prognosis of cancers.

## Background

Cancer has long been a major threat to human health and age, and it is a complex disease, is often characterized by abnormal and uninhibited cell growth (Eeghen et al., 2015; Mcguire et al., 2015; Taber et al., 2016). Unhealthy living environment leads to delay in the repair of damaged body cells, which accumulate with increase in individual's chronological age, thus greatly increasing the risk of cancer (Palmer et al., 2018). Although it has been confirmed that there is a general correlation between individual age and the occurrence of cancer (Zinger et al., 2017), the degree of this correlation may be different due to the age distribution of different tumor sites.

More and more diagnostic, recurrence and prognostic biomarkers of cancer have been identified (Zhou et al., 2015a, 2016). Many age-related biomarkers such as DNA methylation, telomere length changes, cell metabolic factor regulation, transcription level, and protein expression level have been discovered and further studied (Aubert and Lansdorp, 2008; Vilchez et al., 2014; Zubakov et al., 2016). DNA methylation could modulate gene expression during development and cancer progression (Wang et al., 2013; Zhang et al., 2019). In comparison to normal somatic tissues, the cancer methylome is typically characterized by a pattern of global hypomethylation coupled with site-specific promoter hypermethylation (Urbano et al., 2019). Through comparative analysis of a variety of existing age-related biomarkers, DNA methylation has been found as one of the most promising biomarkers for age prediction. DNA methylation refers to the addition of a methyl group to C at the 5th position of cytosine to promote or inhibit gene expression (Dirk, 2015; Nwanaji-Enwerem et al., 2018). In recent years, DNA methylation age (m-age) biomarkers has been able to accurately estimate the age of any tissue throughout life (Horvath, 2013). In addition, m-age biomarkers are valuable tools for evaluating tumor process, which can be a predictor of human health (Perna et al., 2016; Dhingra et al., 2018). However, the differences between m-age and chronological age of different tissues in tumor and the biological processes involved remained to be studied.

There are many studies on the age of individuals using DNA methylation level (Horvath, 2013; Galamb et al., 2016; Dhingra et al., 2018). In 2013, it was proposed for the first time to construct human multi-tissue age predictor based on DNA methylation to measure the degree of aging in human (Horvath, 2013). Subsequently, Weidnei et al. constructed age predictors using three CpG sites as characteristics in 575 healthy samples (Weidner et al., 2014; Nwanaji-Enwerem et al., 2018). Zbiec-piekarska et al. used 5 CpG loci as characteristics in 420 healthy samples to predict age (Spólnicka et al., 2018). Due to tissue specificity of methylation level, Stubbs et al. proposed to construct a multi-tissue age predictor based on methylation level in biological mice model (Stubbs et al., 2017). In addition, DNA methylation is maintained throughout life course in multiple tissues, linking many known early life factor to cancer risk (Kristina et al., 2015). The changes in DNA methylation with age occur at regulatory regions and contribute to tumor development (Johnson et al., 2017). Methylation of SLFN11 is a biomarker for poor prognosis in colorectal cancer and methylations of SLIT1, SLIT2, and SLIT3 are abnormal in gastric cancer. F2RL3 methylation is recently identified as a biomarker closely reflecting both current and past smoking exposure, causing lung cancer (Yan et al., 2015; Kim et al., 2016; He et al., 2017). However, the difference between the m-age for cancer and healthy samples is still unknown. In addition, the effect of age-related methylation characteristics on the survival risk of diverse cancer needs to be further confirmed.

In the present study, we found that DNA methylation levels were correlated with chronological age, and age-associated methylation sites were identified in human cancers. A comprehensive age prediction model named CancerClock was constructed using these age-associated methylation sites. Further, the characteristics of these age-associated methylation sites were described from three main aspects, including GO functions, biological phenotype, and the genomic location of the feature. Moreover, the m-age of common types of human cancers was described. The differences between predicted m-age and chronological age were also identified using CancerClock. For each cancer type, we detected a series of methylation sites, which could influence m-age compared to healthy samples. Finally, these differences were analyzed using cox regression model, and their effect on tumor survival risk was analyzed. In summary, the findings of this study would provide assistance to depict the age clock of cancers, characterize the m-age of cancer samples, and clinically evaluate the cancer progression.

## Methods

### Clinical and Methylation Profile of Cancers

The methylation profile of IlluminaMethylation450 chip for 33 cancer types and their matched normal tissues were obtained from the cancer genome atlas (TCGA) data portal (TCGA Release 14.0, https://portal.gdc.cancer.gov/) (Hutter and Zenklusen, 2018), and each sample contained 485,512 methylation sites. The values in the methylation profile represent methylation degree of the sites ranging from 0 to 1, which is calculated as the ratio of methylated signal. The sample age ranged from 14 to 89 years. Totally, 8,692 samples including 7,988 tumor samples and 704 normal tissue samples were extracted for the present study (Supplementary Table S1). The corresponding clinical information of the samples (including age, gender, survival prognosis, cancer stage, and type of tumor samples) was also described.

### Methylation Data Processing

First, we calculated the mean, standard deviation, and maximum and minimum values of age for each cancer type sample without clinical information were excluded. Methylation sites with NA values >10% in samples were removed. Then, we imputed the remaining NA values using 10-nearest neighbor method with the function knnImputation in DMwR package by R. DNA methylation profiles of normal and tumor samples were annotated using CpG probe annotation file from GENCODE (Release 29, https://www.gencodegenes.org) (Frankish et al., 2019). All processes were performed by R software (R 3.3.3).

### The Segmentation and Statistics of Age for All Cancer and Normal Samples

Human age in this study was divided into four sections according to the novel age subsection proposed by the United Nations and World Health Organization (WHO) after 1994: (Eeghen et al., 2015) Young people: under 44 years old (Taber et al., 2016), middle-aged people: between 45 and 59 years old (Mcguire et al., 2015), older-young people: between 60 and 74 years old, and (Palmer et al., 2018) old people: over 75 years old (Supplementary Table S2). The chronological ages of samples were obtained from clinical information and were divided using above age segmentations rules (Supplementary Table S3).

### Identification of Age-Associated Methylation Sites

To identify age-associated methylation sites, Spearman correlation coefficients (SCCs) were calculated based on age and methylation levels based on all the normal samples (Stubbs et al., 2017). Then, a series of methylation sites were obtained with *P* < 0.05 and |SCC|>0.3. These methylation sites were proved to be significantly correlated with age, and can be the candidate features for constructing the model.

### Construction of Age Predictor Known as CancerClock Based on Methylation Level in Normal Samples

To predict human age, we performed LASSO regression model in the generalized linear mode, which through some regression coefficients was strictly set to 0 and we obtained a model with good performance and strong explanatory power (Hepp et al., 2016). Firstly, we obtained a set of M^*^N methylation matrix X, in which the value in the matrix is the methylation level of the methylation site M in N samples. Each row of X represented the 1,631 age-associated sites, and the column represented the normal samples. Then, we normalized the methylation values as follows:

$$\begin{array}{l} \overset{\text{m}}{\underset{\text{i}=1}{\sum }}{\text{x}}_{\text{ij}}=0,\overset{\text{m}}{\underset{\text{i}=1}{\sum }}{\text{x}}_{\text{ij}}^{2}=\;1 \end{array}$$

From the formulae, i = 1,2,3…m, j = 1,2,3…n, m represents the 1,631 age-associated sites, n represents the sample size, and *x*_*ij*_ represents the methylation level.

Next, we assumed a set of linear regression models:

$$\begin{array}{l} \text{mAge}=\beta_{0}+\beta_{1}{\text{x}}_{1}+\beta_{2}{\text{x}}_{2}+\beta_{3}{\text{x}}_{3}\ldots +\beta_{\text{m}}{\text{x}}_{\text{m}} \end{array}$$

mAge is the model prediction age, vector β is the model coefficient, and vector x is the methylation expression level.

RSSs (residual sum of squares) with the least square estimation:

$$\begin{array}{l} \text{RSS}=\overset{n}{\underset{\text{i}=1}{\sum }}{\left({\text{y}}_{\text{i}}-\beta_{0}-\overset{\text{p}}{\underset{\text{j}=1}{\sum }}\beta_{\text{j}}{\text{x}}_{\text{ij}}\right)}^{2} \end{array}$$

I (*n* = 1, 2, 3…*n*) represents the sample size, y_i_ describes the actual age of sample i, and x_ij_ represents the methylation level. The smaller the RSSs, the better the fitting effect.

LASSO-minimality:

$$\begin{array}{l} \text{J}\left(\beta \right)=\overset{\text{n}}{\underset{\text{i}=1}{\sum }}{\left({\text{y}}_{\text{i}}-\beta_{0}-\overset{\text{p}}{\underset{\text{j}=1}{\sum }}\beta_{\text{j}}{\text{x}}_{\text{ij}}\right)}^{2}+\gamma \overset{\text{p}}{\underset{\text{j}=1}{\sum }}|\beta_{\text{j}}|=\text{RSS}+\gamma \overset{\text{p}}{\underset{\text{j}=1}{\sum }}|\beta_{\text{j}}| \end{array}$$

N means the total number of samples. $\gamma \overset{p}{\underset{j=1}{\sum }}|\beta_{j}|$ represents compressed penalty term. γ means adjustment, which controls the influence of RSS and compressed penalty term on regression coefficient. β_0_ is excluded from the compressed penalty term, which represents the mean of response variables when *x*_*j*_ = 0. Next, coordinate axis descent method was used to solve the model. The initial β values were all 0. When the βvalue was lower than the threshold, we set the two adjacent iterations; the vector β_*k*_ would be regarded as the final feature of our model.

The CancerClock model was constructed based on GLMNET package in R 3.3.3 (Friedman et al., 2010). We evaluated the stability and accuracy of the model by means of 10-fold cross-validation. All the healthy samples were randomly divided into 10 subsets, 9 of which were selected as the training set. The test set was used in the verification of the model.

### Functional Analysis and Phenotypic Traits for Characteristics in CancerClock

With the Enrichr tool online web server (http://amp.pharm.mssm.edu/Enrichr) using default parameters, functional enrichment was performed for genes across core clusters (Kuleshov et al., 2016), and we obtained enriched GO terms (*P* < 0.01, FDR < 0.05). The degree of methylation at chromosomal sites affected post-translational modification of proteins, which in turn contributed to the expression of biological traits. In this study, EWAS atlas, which is online analysis software, was used in describing the selected 282 loci with biological traits. EWAS Atlas (http://bigd.big.ac.cn/ewas) (Li et al., 2019) is a database of epigenetic traits, which include the correlation of information between nearly 330,000 methylation sites and 305 different biological traits through the integration and analysis of existing literatures.

### Characteristics of Genome Location Regions for Age-Related Methylation Sties in CancerClock

To explore the distribution of CancerClock features in genome, we got the annotation information of the genome region from GENCODE database (Release 29, https://www.gencodegenes.org) and we mapped the methylation features on the genome using BEDTools (V2.29.0, https://bedtools.readthedocs.io/en/latest/content/overview.html). In addition, we defined the promoter region as the upstream and downstream 1.5 kb of TSS (transcription start site); thus, the regions included promoter, exon, 5′-UTR, 3′-UTR, and TSS.

### Prediction of m-Age for Tumor Types

To predict human methylation age, we used 282 age-related methylation characteristics and 704 healthy samples to construct age prediction model. To investigate further whether tumor causes changes in methylation age, we used the CancerClock prediction model to predict the 33 cancer types in TCGA based on methylation level. For cancer samples, their methylation age and chronological age showed some difference. Among the age prediction of cancer samples, we defined the error value as the absolute age difference of the quarter-point after the age prediction rank, and we showed the difference between the biology age and methylation age for different cancer types. Thus, some age-influenced cancer samples, in which methylation age was significantly different from chronological age, were obtained.

### Differential Analysis of Methylation Sites Between Normal and “Age-Related Cancer Samples”

To determine which age-related methylation sites were changed in tumor samples, we conducted *t*-test for the methylation sites in healthy samples and age-influenced cancer samples, and we obtained significantly differential methylation sites (FDR < 0.01, Fold Change value>2 or <0.5). These cancer samples were defined as “age-related cancer samples” and they have some differential methylation sites.

### The Survival Analysis for Differential Methylation Sites

An integrated pipeline was constructed to explore the associations between these age-related differential methylation sites and cancer survival (Zhou et al., 2019). First, we divided the samples into two groups including hyper-methylated and hypo-methylated sample groups according to the median methylation level. Next, the age-related differential methylation sites of the 16 cancer types were selected to establish a cox risk regression model for each tumor type, which was selected according to *P* < 0.05 (Gellar et al., 2015). The covariance matrix was composed of the cancer stage, gender, age, and methylation level. Next, the risk score for each cancer patient was calculated according to the linear combination of the methylation values weighted by the coefficient from multivariate Cox regression analysis. The median risk score was used as the cut-off point to divide the cancer patients into high and low risk groups. Finally, Kaplan-Meier survival analysis was conducted for the two groups, and statistical significance was assessed using the log-rank test. The survival results were considered significant when *P* < 0.05. All analyses were performed within the R 3.3.3 framework.

## Results

### Correlation Between the Levels of Some DNA Methylation Sites in Human Normal Tissues and Chronological Age

**Figure 1:**
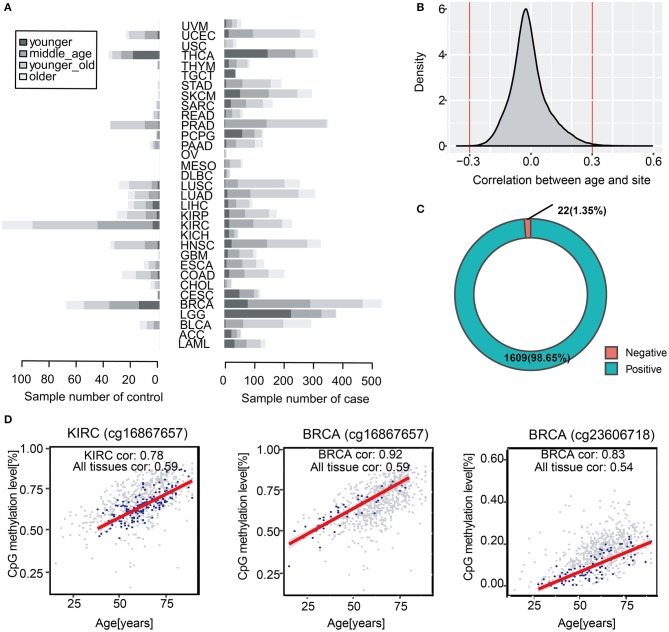
Identification of chronological age-related DNA methylation sites in human normal tissues. **(A)** The barplot shows sample distribution of four age segments in diverse cancer types. The lighter color represents larger age of samples. **(B)** Distribution of correlation scores between all methylation sites and chronological age. **(C)** Spearman correlation between age and methylation (*P* < 0.05, |cor| > 0.3). The pie chart shows the proportion of 1,631 positive and negative methylation sites correlated with chronological age. **(D)** The scatter diagram shows the correlation between methylation sites (cg16867657, cg23606718) and chronological age in KIRC and BRCA. The overall background is the methylation sites of all samples (gray color), and the blue color represents the correlation between chronological age and methylation age in a certain type of samples such as BRCA and KIRC.

Chronological age is referred to the real age of an individual, which is recorded by researchers. To study age-associated methylation levels in human over a wide range of ages and tissues, we collected 704 normal tissue samples from 14 to 89 years (Figure 1A). We found that 145,523 DNA methylation sites across all tissues were correlated with age (a multiple testing corrected *p* < 0.05) (Figure 1B). Further, we screened the correlations and we obtained 1,631 methylation sites that were significantly correlated with age, which were considered to be significantly correlated with chronological age (|cor| >0.3). Overall, we identified up to 1,609 positive correlations, accounting for 98.7% of the total number of methylation sites, which suggested that in most samples, the higher the level of age-related methylation, the older the organism in the sample (Figure 1C). We classified tissue samples follow tissues to verify tissue specificity of the 1,631 age-related sites stated above. We performed correlation analysis with chronological age of each tissue, and we compared them with all-tissue correlation scores. The correlations of the most specific tissues were higher than the overall correlation level. This indicated that the selected sites were not only significantly correlated with age in all samples, but also more highly correlated with chronological age of samples in a single tissue (Figure 1D).Therefore, we demonstrated that these 1,631 methylation sites were related to the chronological age of individuals not only in specific tissues but also in all samples.

### Feature Selection and Model Construction of CancerClock

**Figure 2:**
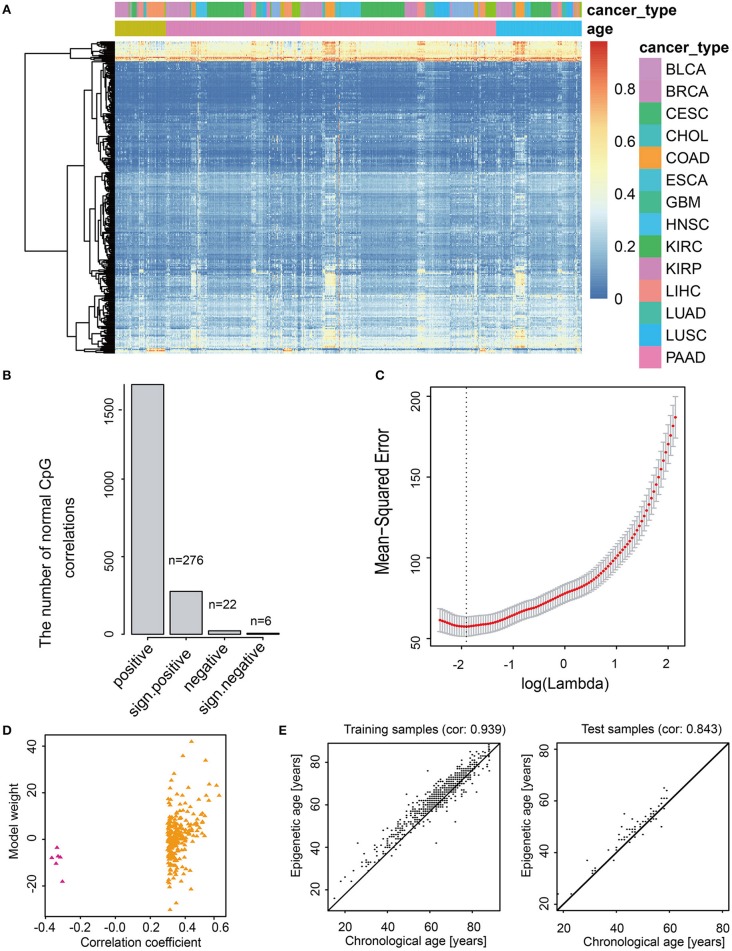
Feature selection and model construction of CancerClock. **(A)** The heatmap shows the 282 age-related methylation sites extracted by the model in all cancer samples. The red and blue represent high and low levels of methylation sites. **(B)** The barplot shows the number of 282 positive and negative age-related methylation sites. **(C)** The selection of thresholds in LASSO regression model. The line represents the coefficient values, and the minimum mean-square error corresponding to log(Lambda) is−1.95197. **(D)** Scatter diagram shows the correlations between correlation coefficient and model weight of CancerClock. **(E)** The correlation between chronological age and predicted m-age in the training set and the correlation between chronological age and predicted m-age in the test set.

CancerClock model was constructed to predict the m-age in human normal samples based on adjacent normal tissue for each cancer (Figure 2A, see Methods). The LASSO linear regression model was used to screen the 1,631 age-related methylation sites in the training set using the GLMNET package of R. Finally, 282 sites were selected from 1,631 methylation site as model features (Figure 2B, Supplementary Table S4). According to the least Mean-Squared Error (MSE, reflect the degree of difference between the estimator and true value, the smaller the MSE, the better the model fit) of LASSO linear regression model, we found that the value β_0_ of the model was 34.63 when the adjustment parameter was 0.1419941 (Figure 2C). Among the 282 methylation characteristics in the construction of model, the levels of cg08461576, cg05923914, cg27641628, cg13221458, cg05632420, and cg07103722 were significantly and negatively correlated with sample age, and the linear model coefficients of the six sites above were negative as well (Figure 2D). We described the methylation level for these 282 methylation characteristics and we promoted them to effectively describe the chronological age of all normal samples through the heatmap (Figures 2A,D). The results showed that it was difficult to characterize the methylation age of samples by single methylation sites, and the methylation clusters might produce a better result.

To evaluate the accuracy of the model, we used the training set and the test set to verify the model, separately. The results showed that the model responded well to the training set samples, and the correlation between chronological age and m-age was as high as 0.939 (*P* < 2.38e−295) (Figure 2E). Meanwhile, for test set samples, the correlation between chronological age and m-age predicted by the model was up to 0.843 (*P* < 5.96e−20) (Figure 2E). The average accuracy of the model was 81%. To determine the difference between chronological age and predicted m-age in normal samples, we ordered the absolute value of the difference between the chronological age and predicted m-age in descending order, and we selected the quartile as the error value. The results showed that the age error value was 3 years, which indicated that the prediction error of over 75% of the sample age was controlled within 3 years, which is far lower than the age error predicted by previous researchers through telomeres.

### Biological Processes and Phenotypic Traits of Age-Related Methylation Sites for CancerClock

**Figure 3:**
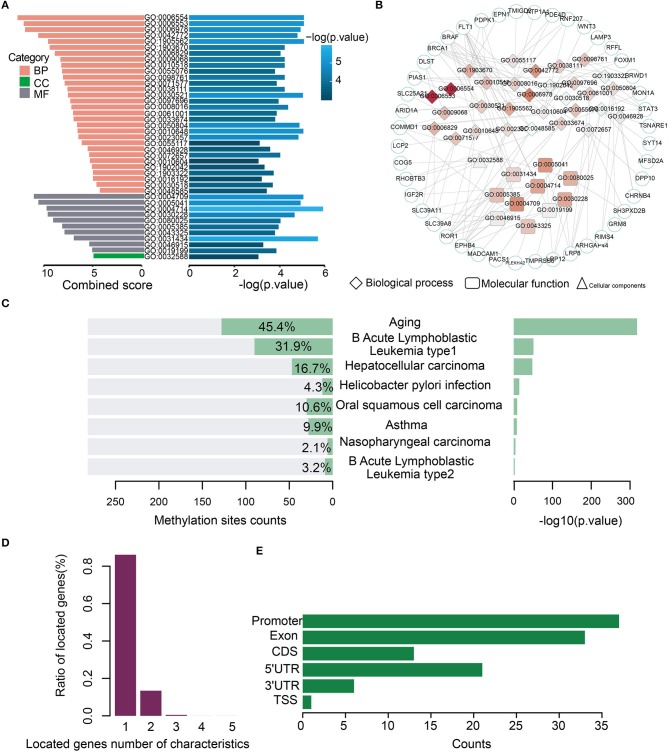
Biological processes and phenotypic traits of age-related methylation sites for CancerClock. **(A)** The barplot shows the combined score and –log (*p*-values) for enrichment GO terms of 282 extracted methylation sites. Pink, green, and gray colors represent biological process (BP), molecular function (MF), and cellular component (CC), respectively. **(B)** Network shows the interactions between GO terms and genes. The color of node in network indicates enrichment strength, and the three different shapes represent different biological types of GO terms. The circle represents the genes. **(C)** The bar chart shows the enrichment counts and significance *P*-values of each trait from EWAS atlas analysis. **(D)** The corresponding relationship between methylation site and the genes in which it is located. The relationship is usually one-to-one or one-to-many. **(E)** The distribution of 282 features of CancerClock model in genome position.

Gene Ontology (GO) analysis of the genes for the 282 model characteristics revealed that these genes were associated with some GO terms such as “lysine catabolic process” (GO:0006554) and “lysine metabolic process” (GO:0006553) (Figure 3A). A network consisting of GO terms and genes was constructed (Figure 3B). These results indicated that age-related methylation sites could alter many important biology processes (Supplementary Table S5). The online analysis software of EWAS Atlas was used to analyze the biological characteristics enrichment of 282 selected features, and it was found that 128 of them were significantly enriched in age traits [–log10 (*p*) > 318], which was manifested as significant methylation quantitative trait loci (meQTLs) related to age. Meanwhile, 128 sites were enriched in age ontology entry (GO:0007568). In addition, we found that most methylation sites were enriched in human acute leukemia [–log10 (*p*) > 50] (Figure 3C, Supplementary Table S6). When we mapped these characteristics on the corresponding gene positions, we found that most of the loci corresponded to a single gene, while few loci corresponded to multiple genes (Figure 3D). To describe the distribution of these features of CancerClock in genomic regions, we determined the position of these features in the genome. We found 37 of the 282 characteristics located in the promoter region of genes (Figure 3E).

### Predicted m-Age and Chronological Age of Cancer Patients

**Figure 4:**
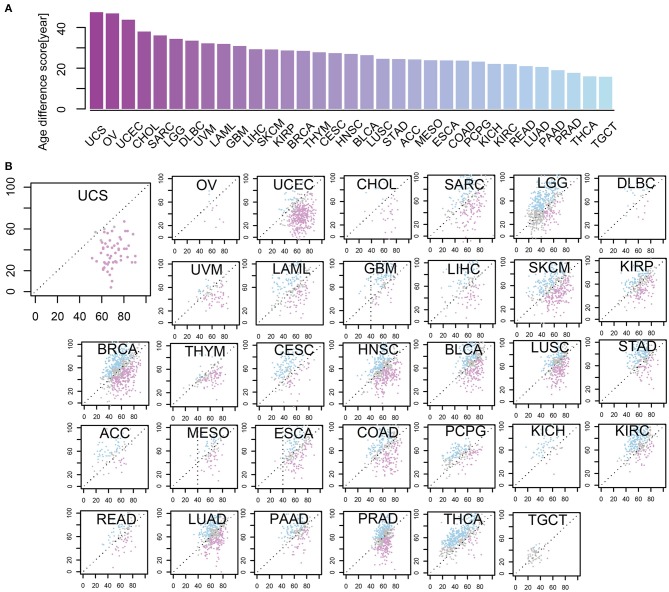
The levels of some methylation sites were differential in age-related cancer samples between m-age and chronological age. **(A)** Age different scores between chronological age and predicted m-age among 33 cancer types. **(B)** The relationship between chronological age and predicted m-age for all the 33 disease types. Compared to the chronological age group, the pink color indicates that the predicted m-age group was down-regulated, the blue color indicates that the predicted m-age group was up-regulated, and the gray color indicates that the predicted m-age group remained unchanged.

To depict the difference between m-age and chronological age in cancer samples, we utilized the CancerClock based on the methylation levels of all the 33 tumor types. We found that the m-age was generally different from the chronological age, but the degree of the difference was closely related to tumor types. Here, we ranked the absolute age differences between chronological age and we predicted the m-age and selected the quartile values as the age difference scores of tumors, separately (Figure 4A, Supplementary Table S7). A number of tumors showed higher age difference score. Uterine carcinosarcoma (USC) had the highest age difference score, followed by ovarian serous cystadenoma carcinoma (OV) and uterine corpus endometrial carcinoma (UCEC). We found that the top three diseases with the largest age difference scores were all women tumors, suggesting that their chronological age is related to the pathological changes of the reproductive organ, and that female diseases affect chronological age to some extent. In addition, the distribution of m-age compared to the chronological age was described, separately (Figure 4B). We found that the m-age of different disease types was specific to their chronological age. For example, USC showed that all the 57 cancer samples were younger than the chronological age, and 96% of 54 testicular tumors showed age decline in men. According to our results, in the 121 and 184 samples with thymoma and ganglioma, 95% of the m-age was below the chronological age, respectively. In addition, in the 247 and 80 samples with renal cell carcinoma (RCC) and melanoma, 94% had younger m-age than chronological age, respectively.

### Differential Levels of Some Methylation Sites Between m-Age and Chronological Age in Age-Related Cancer Samples

To explore further the difference between m-age and chronological age, we selected 25% age-related samples with the largest difference, and then performed differential analysis with normal samples in each cancer type (Supplementary Table S8). In the age-related samples of cervical squamous cell carcinoma and endocervical adenocarcinoma (CESC), there were 77 methylation sites levels that were, respectively, different (FDR < 0.01, |log (FC)|>1) (Supplementary Table S9). Moreover, there were 38 methylation sites with significant changes in breast invasive carcinoma (BRCA) and 22 methylation sites with significant changes in kidney renal clear cell carcinoma (KIRC). The differential methylation sites of some cancers showed hyper-methylation in tumor samples as follows: cervical squamous cell carcinoma (CESC) and bladder urothelial carcinoma (BLCA), breast invasive carcinoma (BRCA), colon adenocarcinoma (COAD), kidney renal clear cell carcinoma (KIRC), lung squamous cell carcinoma (LUSC), pancreatic adenocarcinoma (PAAD), sarcoma (SARC), and uterine corpus endometrial carcinoma (UCEC). It is suggested that hyper-methylation of these age-related differences may lead to changes in m-age and cancer development.

### Association Between the Differential Methylation Sites in Age-Related Samples and Survival

**Figure 5:**
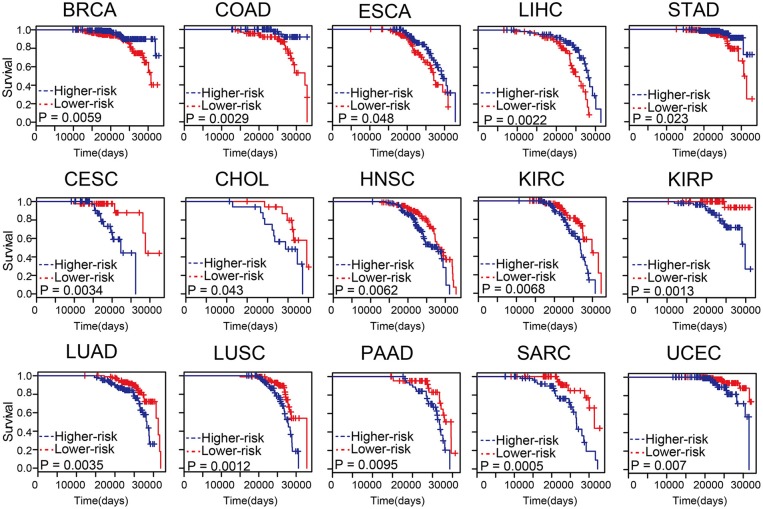
The differential methylation sites in age-related samples were associated with survival. Kaplan-Meier survival analysis of two groups of patients with high- (blue line) and low- score (red line) groups. Survival days are shown along the X-axis. Overall survival rates are shown along the Y-axis.

Predicting survival state of cancer patients was critical and challenging (Zhou et al., 2017; Bao et al., 2019). To evaluate the influence of the methylation level on patient survival, cox risk regression model was performed for the differential methylation sites in age-related samples of each tumor type (Supplementary Table S10). The patients were divided into two groups according to median risk score. In most cancer types, these differential methylation sites in age-related samples were associated with survival (Figure 5). The higher the risk scores of BRCA, COAD, esophageal carcinoma (ESCA), liver hepatocellular carcinoma (LIHC), and stomach adenocarcinoma (STAD), the lower the survival of the patients. The results indicated that differential methylation sites of age-related samples maybe could be an effective prognostic biomarker for cancers.

## Discussion

DNA methylation levels were closely associated with age and related to the occurrence of tumors (Wang et al., 2016). In this study, we explored the correlations between DNA methylation level and age in normal tissues. Importantly, we established CancerClock, a predictive model of age based on DNA methylation level to depict the age clock in tumors. This predicted the age based on methylations of 282 sites from different tumor samples and it allowed us to access the m-age among methylation datasets. In addition, we found that the model was affected by a variety of biological processes, which may indicate the molecular influence of methylation on age. Age difference score in tumor samples showed the extent to which age affected different tumors. Meanwhile, methylation sites affecting the m-age of these tumors were identified. Through weighted survival analysis of cancer samples, we finally determined the impact of these age-causing sites on tumor survival.

CancerClock model is a multi-tissue age prediction model based on the methylation level of diverse cancer types in TCGA. It adopted a similar way as Horvath clock in building the age prediction model of normal samples (Horvath, 2013). More importantly, we applied this model to cancer samples and we depicted the difference between m-age and chronological age. These differences between m-age and chronological age may provide assistance to understand cancer development. Previous study also showed that epigenetic age acceleration is associated with colorectal cancer molecular characteristics and can be a significant predictor of overall survival, as well as age and tumor stage (Zheng et al., 2019). DNA methylation-based measures of biological age may be an important predictors of breast cancer risk (Kresovich et al., 2019). In the present study, we depicted this phenomenon in multiple cancer types and we tried to explain the mechanism by which methylation levels contribute to m-age and cause cancers.

Many studies used expression of gene and lncRNA to predict cancer development and prognosis (Zhou et al., 2015b, 2018; Sun et al., 2019). In present work, m-age was considered as cancer biomarker for development and prognosis. CancerClock could be applied in predicting biological age for normal samples. The differences between biological and m-age were also could be identified. These differences could be used to explore the roles of methylation in cancer development and prognosis. In future work, more samples and experiments should be used to validate the present work.

In summary, the present study suggested that some methylation sites were associated with chronological age. Comprehensive age predicator CancerClock could predict m-age for normal samples and could find the differences between m-age and chronological age in age-related cancer samples. We further discovered the differential methylation sites between age-related cancer samples and normal samples. These differential methylation sites were associated with survival in cancers. In addition, the present study suggested that DNA methylation-based measures of chronological age might be important predictors of cancer risk.

## Data Availability Statement

All datasets generated for this study are included in the article/Supplementary Material.

## Author Contributions

SN, LW, and HC conceived the project. TZ, YG, JW, and XL acquired the data. SS, YW, and SG analyzed the data. HZ, HL, and DS constructed the data resource. TZ, SN, and YG wrote the manuscript. All authors read and accepted the final version of the manuscript.

### Conflict of Interest

The authors declare that the research was conducted in the absence of any commercial or financial relationships that could be construed as a potential conflict of interest.

## Abbreviations

m-age: DNA methylation age
TCGA: The cancer genome atlas
MSE: Mean-squared error
GO: Gene Ontology
EWAS: epigenome-wide association study software
BLCA: Bladder urothelial carcinoma
BRCA: Breast invasive carcinoma
COAD: Colon adenocarcinoma
ESCA: Esophageal carcinoma
HNSC: Head and neck squamous cell carcinoma
KICH: Kidney chromophobe
KIRC: Kidney renal clear cell carcinoma
KIRP: Kidney renal papillary cell carcinoma
LIHC: Liver hepatocellular carcinoma
LUAD: Lung adenocarcinoma
LUSC: Lung squamous cell carcinoma
PRAD: Prostate adenocarcinoma
READ: Rectum adenocarcinoma
STAD: Stomach adenocarcinoma
THCA: Thyroid carcinoma
UCEC: Uterine corpus endometrial carcinoma
CHOL: Cholangiocarcinoma
OV: Ovarian serous cystadenocarcinoma
GBM: Glioblastoma multiforme
PAAD: Pancreatic adenocarcinoma
CESC: Cervical squamous cell carcinoma
PCPG: Pheochromocytoma and paraganglioma
SARC: Sarcoma
THYM: Thymoma
SKCM: Skin cutaneous melanoma
ACC: Adrenocortical carcinoma
CCSK: Clear cell sarcoma of the kidney
DLBC: Lymphoid neoplasm diffuse large B-cell lymphoma
MESO: Mesothelioma
LGG: Brain lower grade glioma
TGCT: Testicular germ cell tumors
UCS: Uterine carcinosarcoma
UVM: Uveal melanoma.
